# Supplementary material for: Toxicokinetics of Hydrolyzed Fumonisin B1 after Single Oral or Intravenous Bolus to Broiler Chickens Fed a Control or a Fumonisins-Contaminated Diet
Source: Toxins (Basel). 2020 Jun 21;12(6):413. doi: 10.3390/toxins12060413 (PMC7354465; doi:10.3390/toxins12060413)
Supplement: Supplementary file 1 [file toxins-12-00413-s001.pdf]

Supplementary Materials: Toxicokinetics of Hydrolyzed Fumonisin B1 After Single Oral or Intravenous Bolus to Broiler Chickens Fed a Control or a Fumonisins-Contaminated Diet

Gunther Antonissen, Siegrid De Baere, Barbara Novak, Dian Schatzmayr, Danica den Hollander, Mathias Devreese and Siska Croubels

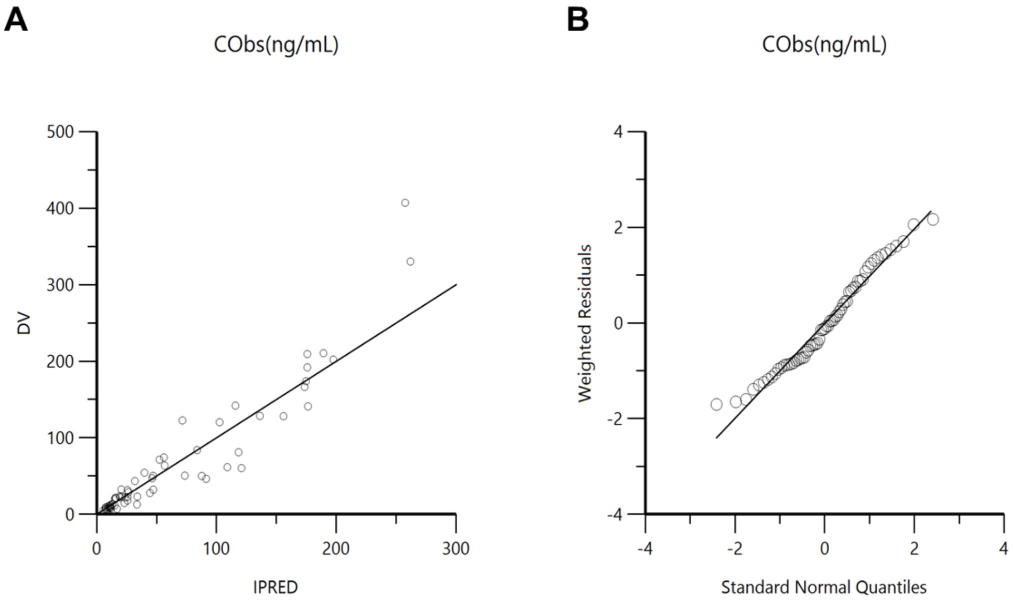

Figure S1. Visual evaluation of the population model of HFB1 after intravenous (IV) dosing: scatter plot of the population dependent variable (DV), namely observed plasma concentration (C_{obs}), versus the individually predicted plasma concentration values (IPRED) (**A**) and QQ plot of the conditionally weighted residuals of C_{obs} (**B**).
